# Rapid qualitative analysis in a mixed-methods evaluation of an infection prevention intervention in a UK hospital setting during the COVID-19 pandemic: A discussion of the CLEAN study methodology

**DOI:** 10.3389/fsoc.2022.958250

**Published:** 2022-10-25

**Authors:** Ruchi Higham, Simon Pini, Aaron Quyn, Mikolaj Kowal, Jack Helliwell, Razan Saman, Penny Lewthwaite, Nicola Young, Nikki Rousseau

**Affiliations:** ^1^Leeds Institute of Clinical Trials Research, University of Leeds, Leeds, United Kingdom; ^2^Leeds Institute of Health Sciences, University of Leeds, Leeds, United Kingdom; ^3^Leeds Institute of Medical Research, University of Leeds, Leeds, United Kingdom; ^4^Leeds Teaching Hospitals Trust, Leeds, United Kingdom

**Keywords:** rapid analysis, rapid evaluation, rapid qualitative research, rapid appraisal, rapid research, infection prevention and control, surface cleaning

## Abstract

The COVID-19 pandemic created an urgent need for high-quality rapid research. One clinical challenge was how to minimise the risk of transmission in the hospital setting. The CLEAN study conducted a rapid evaluation of the potential utility of a spray-based disinfectant in a hospital setting. The study was undertaken between December 2020 and March 2021 and involved the implementation of the spray in 10 different clinical areas in one UK teaching hospital. A mixed-methods approach was adopted (including observations, surveys, and qualitative interviews) informed by the theories for understanding the implementation of new healthcare technologies. The evaluation found that while the spray had a number of perceived benefits when added to existing disinfection processes, other factors limited its potential utility. These findings informed a number of recommendations for future adoption within hospital settings. This paper describes and reflects on the rapid methodology that allowed us to undertake the study and deliver results in a short space of time. We experienced a number of pressures during set-up and fieldwork due to the challenging conditions caused by the pandemic, and the methodological approach had to evolve throughout the study because of the changing clinical context. The involvement of clinicians from the research setting as full members of the research team was key to the rapid delivery of the research. They provided an essential link to the implementation environment, and their experiential knowledge of the setting added an important perspective to the analysis. Balancing their involvement with their clinical roles was challenging, however, as was coordinating a large and diverse team of interviewers in such a short space of time. Overall, the study highlighted the value of rapid research to inform urgent healthcare decisions in a pandemic. Although our experience suggests that conducting such research requires some practical and methodological trade-offs, we found that there were also numerous benefits of using rapid methods and identified various opportunities to ensure their robustness.

## Introduction

The onset of the COVID-19 pandemic presented unique challenges to healthcare systems. One important clinical challenge was how to minimise the risk of transmission in the hospital setting while keeping infection prevention and control (IPC) procedures manageable. Effective IPC procedures are critical to protecting healthcare workers, reducing hospital-acquired infections and preventing onwards transmission to the general population. Research on healthcare workers' (HCWs) experience of IPC during the pandemic has predominantly focussed on the availability and use of personal protective equipment (e.g., Brooks et al., 2021; Hoernke et al., 2021; Broom et al., 2022), and less attention has been paid to surface cleaning and disinfecting.

A spray-based disinfectant was developed by the British Army in the early days of the pandemic to provide protection for its service people. The spray demonstrated efficacy against the COVID-19 virus at a level required by British and European standards for surface disinfectants used in the medical settings (Anderson et al., 2021), and proof of concept field trials conducted by the Army confirmed the spray technology delivered rapid, high-density coverage. The Army was keen to make this technology available in the healthcare setting to help protect patients and healthcare workers. Most surface cleaning in hospitals uses agents with a broad-spectrum of anti-microbial activity that is applied manually, for instance, in the form of a wipe. Manual cleaning can be challenging, time-consuming, and insufficiently thorough (Donskey, 2019), and the spray could potentially address these drawbacks. However, although a formative usability study had been conducted in simulated healthcare environments, there was a lack of evidence about the spray's capability, utility, and acceptability in real-world hospital settings, especially in the context of a novel respiratory virus causing a global pandemic.

The CLEAN study (critical evaluation of the implementation of VIRUSEND in clinical settings) was a rapid evaluation funded under a call for rapid research to address the challenge of COVID-19. A single-centre, prospective implementation study was conducted between December 2020 and March 2021 in a large teaching hospital in the North of England. The overall aim was to assess the utility of the spray in different clinical environments to inform potential wider adoption into routine hospital infection prevention and control processes. The main objectives were to determine the followings: (i) the clinical environments where the spray offers the most potential; (ii) barriers and enablers to implementation at organisational, ward, and individual levels; and (iii) any unintended consequences of implementation.

The rapid evaluation achieved its objectives, reporting clear findings and recommendations, which informed plans for wider adoption. The focus of this paper was to provide a detailed description of, and reflection on, the rapid methodology that allowed us to undertake the study in a short space of time, in line with the reporting guidelines for rapid research proposed by (Vindrola-Padros, 2021, p. 142–147).

## Methods

### Study design

A mixed-methods, rapid evaluation approach (Vindrola-Padros et al., 2021a) was adopted, using surveys, interviews, observations, and key informants to understand the implementation and provide timely results appropriate to the pressurised context of the pandemic. The design was informed by two theories for understanding the implementation of new healthcare technologies: a Framework for Theorising and Evaluating Non-adoption, Abandonment, and Challenges to the Scale-Up, Spread, and Sustainability of Health and Care Technologies (NASSS) (Greenhalgh et al., 2017) and Normalisation Process Theory (NPT) (May and Finch, 2009).

**Figure 1:**
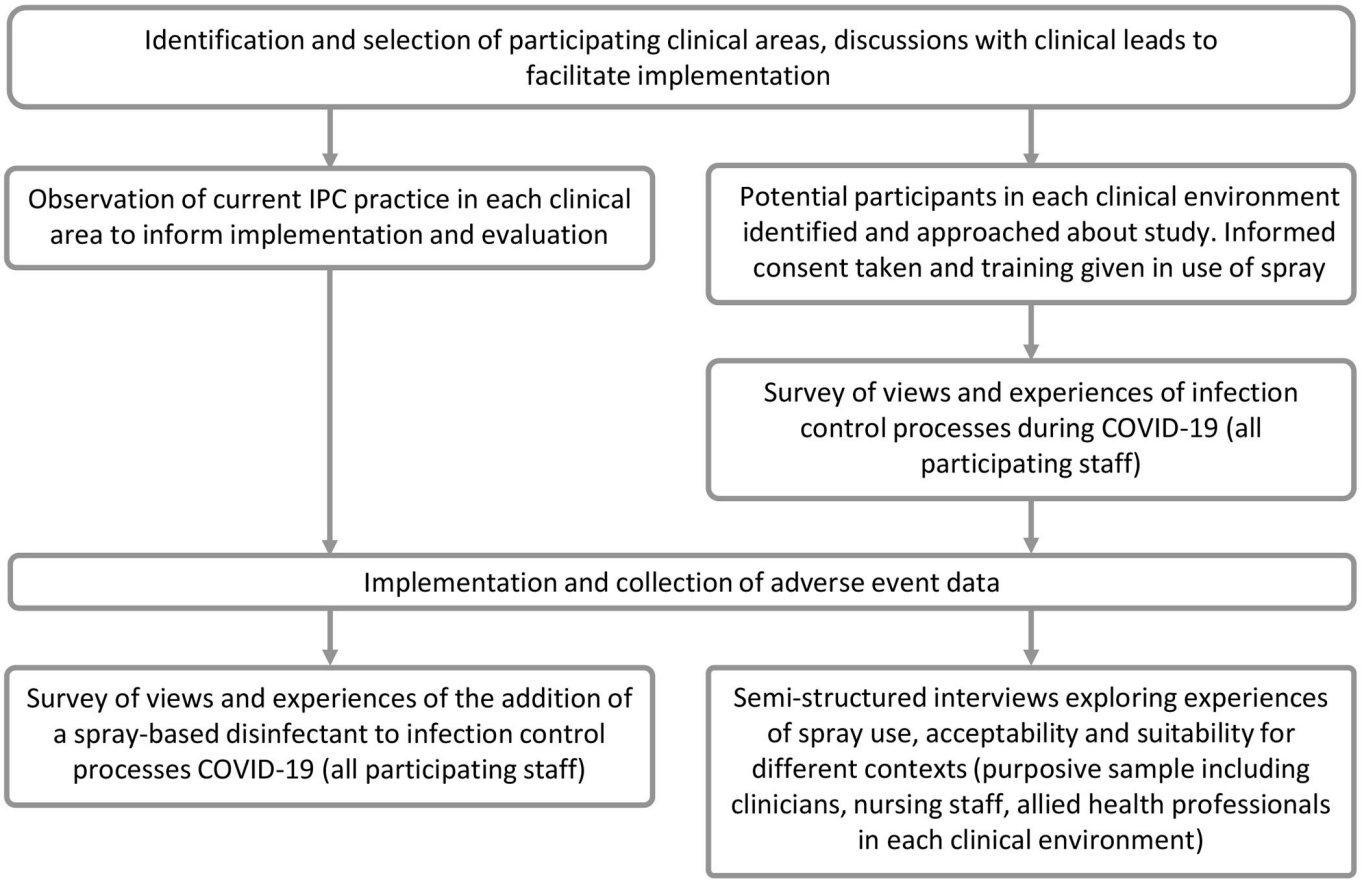
Study flowchart.

**Figure 2:**
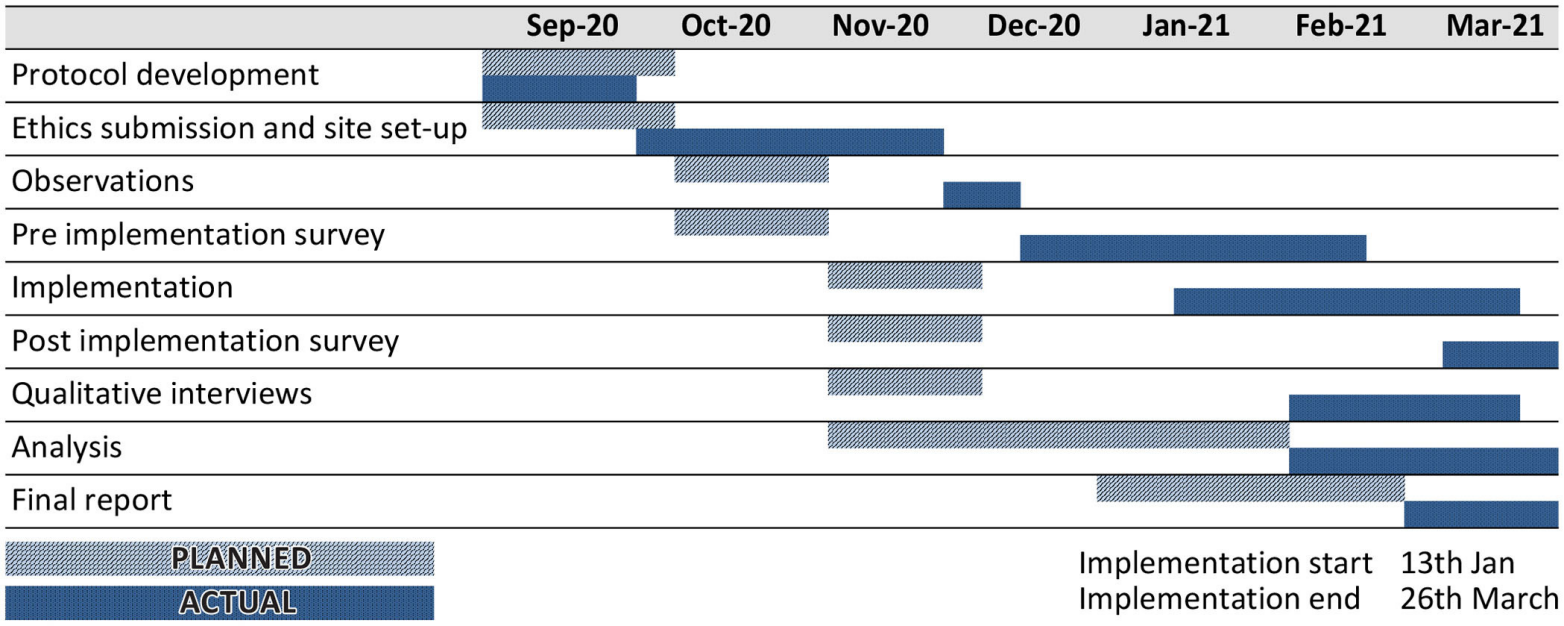
Study timelines.

Figure 1 provides an overview of the study design, and Figure 2 shows the study timelines (both planned and actual). Observations were undertaken prior to an implementation to understand the cleaning processes in participating clinical areas to inform the implementation plan and training materials. The pre-implementation survey was conducted to capture an overview of HCWs' views and experiences of IPC processes, including the perceptions of their own safety in the workplace during COVID-19. This provided context for the evaluation of the spray and also informed the sampling for the qualitative interviews. Qualitative interviews were conducted once a participant had used the spray for a period of time, so that their usage experiences could be explored in depth. The interviews also provided important context about participants' experiences during the COVID-19 pandemic and their views on IPC processes in the hospital generally. This helped interviewers familiarise themselves with the implementation setting, informed probing questions about the use of the spray, and helped to contextualise the analysis of the study findings. The post-implementation survey provided an overview of HCWs' experiences of using the spray across the different clinical areas, including its acceptability and suitability for different contexts, and was also used to interrogate some of the initial findings from the qualitative interviews. The second survey also included an opportunity for those participants who had not completed the initial survey to complete the initial IPC questions *via* a branching question.

### Setting

The study took place in the UK National Health Service (NHS), which provides publicly-funded medical and healthcare services that are free at the point of use for UK residents. Implementation took place in one NHS Trust located in an urban setting in the north of England. The Trust is one of the largest teaching hospitals in Europe, providing healthcare and specialist services for people in the city and surrounding areas. It treats 1.5 million patients every year, including more than 200,000 emergency patients, and employs more than 20,000 staff. Services are provided across seven hospitals and medical services located throughout the city. In total, three of these locations participated in the implementation. Participating clinical areas included outpatient services, theatres, research/administrative activities, and facilities support (e.g., porters).

### Intervention

The spray used in the implementation is manufactured by Pritchard Spray Technology Ltd. It uses compressed air to allow for rapid application over a wide surface area. It can be used on various surfaces, including floors, furniture, and light switches, but at the time of the study had not been approved for the use on medical devices. Prior to the CLEAN study, the spray had been evaluated in a simulated hospital environment, which provided information to support training. Users were advised to apply a fine mist from about “arms' length,” leave for 1 min and then either leave to dry or wipe off as preferred. The spray was initially made available to the staff as a 365-ml bottle; part way through implementation a smaller “pocket-size” 75-ml bottle was also made available.

### Implementation

In total, 10 clinical teams participated in the study, representing a diversity of clinical environments with variation in the level of infection risk (i.e., including areas treating patients with active COVID-19 infection, environments to which patients were only admitted after testing negative for COVID-19, and areas where COVID-19 status was unknown) and different IPC challenges (e.g., areas needing rapid cleansing between patients, offices, and areas where cleanliness was particularly important such as operating theatres). Some teams were strongly associated with a particular location in the hospital (e.g., the Emergency Department) – these teams were typically multi-disciplinary involving different health professionals. Other teams worked across multiple different hospital environments, undertaking a particular task (e.g., porters) or supporting a particular patient group (e.g., patients with cancer) at various stages in their clinical pathway (outpatients, inpatient, surgery, etc.).

Lead clinicians for each environment were approached by the research team, and approval was sought for the use of a spray-based disinfectant to be piloted. The implementation was tailored to each clinical environment; observations of IPC practise were used to inform implementation and training, and scenarios for the use in each clinical environment were approved with necessary stakeholders. Consideration was also given to which equipment the spray could be applied to so as to not invalidate product warranties or breach medical device regulations. Due to the current phase of testing, with evidence only available for efficacy against COVID-19 and not against other infectious agents of concern in a hospital setting, infection control specialists on the project team advised that the spray should only be used as an additional layer of infection prevention and control, rather than replacing other routine and established disinfection processes.

### Recruitment and sampling

All staff members working in participating clinical areas were invited to take part in the implementation. Research nurses approached potential participants in their clinical environments to disseminate study information and gather consent. To maximise efficiency aligned with the rapid methods of the project, research nurses combined gathering consent with providing training on how to use the spray. At this stage, participants consented to participating in the implementation, to providing contact details for receiving the survey invitations, and indicated their willingness to be invited to an interview. Participants were also given a link to an education video, which included an introduction to the project by a senior member of the hospital IPC team, who explained the purpose of the study and provided training in the use of the spray.

We aimed to obtain a purposive sample of participants in the qualitative interviews with attention to the profession, role, seniority, clinical environment, and length of time in the environment. Study recruitment was reviewed at weekly project meetings involving the site and research teams, and interview recruitment was monitored against the purposive sampling characteristics.

### Data collection

#### Observations

Non-participant observations were carried out in-person by three clinical members of the research team (RS, MK, and JH). Approval was sought by the clinical leads before commencing observations. An observation pro-forma (Supplementary Appendix A) was developed by a member of the team with experience of IPC processes (RS) and was used in each environment to understand current IPC procedures and potential gaps where the spray could be used. Findings from this stage informed the implementation strategy.

A limited amount of additional informal participant observation was also conducted to support the implementation. Project team members who participated in meetings to prepare for implementation and research nurses involved in recruitment and implementation made anonymised notes of key points and issues raised by hospital staff, and these were shared and discussed at weekly project meetings. A secure Microsoft Teams site was created to enable rapid sharing of key information among the team. The findings were used to adapt the implementation strategy, tailor training materials, address recruitment challenges, and inform the sample and topic guide for interviews.

#### Surveys

The content of both surveys was developed by the research team (NR, SP, RS, and RH), in consultation with clinical members of the project team, and was informed by the NASSS and NPT frameworks (and, for the second survey, by initial analysis of qualitative interview data). Surveys were distributed to all participating staff and completed electronically using a web-based system (www.onlinesurveys.ac.uk/). Invitations and reminders were sent *via* email, or by text message where an email address had not been provided, and participants were provided with their study number, so they could complete the survey confidentially. Invitations for the pre-implementation survey were sent out shortly after participants consented. Invitations to take part in the post-implementation survey were sent once participants had been using the spray for a period of time (between 2 and 8 weeks, depending on when the participant was recruited).

#### Qualitative interviews

Semi-structured qualitative interviews were conducted following the implementation of the spray. Interviews were conducted using Microsoft Teams and were audio-recorded. Following the interview, both the recording and the auto-transcript were retrieved from Microsoft Teams and the recording was then deleted from the Microsoft Teams space. The topic guide (Supplementary Appendix B) incorporated the key aspects of the NASSS and NPT frameworks and covered the context of implementation (participant's role and any changes during COVID-19; IPC processes in the clinical environment) and views and experiences of using the spray. Alongside using the topic guide, novel areas arising during interviews were explored for relevance and then incorporated into subsequent interviews if the research team considered them worthwhile topics to explore in more detail. Developing themes from the early stages of analysis and survey responses were also explored in later interviews.

Due to the rapid methods being employed, a team of six researchers conducted the interviews, including experienced qualitative researchers (NR, SP, and RH) and junior doctors/clinical fellows with no prior experience of qualitative research (RS, MK, and JH). Junior doctors/clinical fellows interested in obtaining research experience were approached by study clinical co-applicants and invited to join the study team – clinical fellows came from infection control (RS) and surgical (MK and JH) specialties. There was an equal split in gender with three female (NR, RH, and RS) and three male (SP, MK, and JH) interviewers. The doctors all worked in clinical areas participating in the study and did not interview HCWs from their own teams. They were given two training sessions prior to conducting qualitative interviews; these sessions focussed on interview technique and the practical aspects of conducting interviews. They were also provided with opportunities to view interviews conducted by experienced qualitative interviewers and to practise using the CLEAN topic guide.

### Analysis

Quantitative data were downloaded into Microsoft Excel and cross-tabulated by clinical area and key participant characteristics. Data were summarised descriptively, e.g., frequencies (and percentages) or means/medians (and standard deviation/interquartile range).

Qualitative data were analysed using a rapid qualitative analysis approach. Weekly meetings of the qualitative team were held to enable sharing of initial reflections on the interviews and begin discussion of potential analytic categories. To facilitate this oral analysis process, each interviewer completed a “rapid analysis procedure sheet” (RAP sheet) for each participant (Vindrola-Padros et al., 2020b), which summarised the interview content and their initial reflections (Supplementary Appendix C). RAP sheet headings were broadly defined following the first two interviews and then reviewed and adjusted through team reflection and discussion. Towards the end of the interview period, one researcher (SP) retrieved all of the individual participant's RAP sheets and synthesised the themes into a combined thematic framework. The framework was further refined through iterative discussions in the subsequent weekly oral analysis meetings. Each member of the qualitative team then used this framework to systematically search their interviews for quotes related to each of the themes, taking care to identify diverse views in each area. This was done using a combination of notes made during the interviews, interview recordings, and auto-transcripts. The analysis continued to develop throughout the process of preparing final reports and papers.

### Ethical considerations

The study was sponsored by the University of Leeds (UoL) and funded by Innovate UK, part of UK Research and Innovation (grant reference: 77807). The spray manufacturer was an industry partner on the grant and provided supplies of the spray for the study at no cost. The industry partner took no part in data collection or analysis. Ethical approval was granted through by the Frenchay Research Ethics Committee (REC) (20/SW/0178) *via* the expedited approval route for urgent COVID-19 research. Health Research Authority (HRA) approval was granted, and Confirmation of Capacity and Capability was received from the site research governance office. Medicines and Health products Research Agency (MHRA) approval was not required as the intervention is not classified as a medical device. The study was conducted in line with the requirements of the GDPR and the Data Protection Act (2018) with regard to the collection, storage, processing, and disclosure of personal information.

The risks that the research activity posed to IPC within the hospital and the risk of COVID-19 infection to the research team were regularly reviewed at weekly project management meetings. All in-person research activity was conducted by team members who were hospital staff and trained to work in clinical environments during COVID-19 and had received an individual risk assessment. Face-to-face meetings and data collection were kept to the minimum necessary, and social distancing guidelines were followed at all times.

#### Informed consent

Written informed consent was obtained prior to the participants undergoing any data collection. Separate consent was taken for participating in the implementation, surveys, and interviews. For the observation aspect of the study, individuals in the research setting were not deemed to be research “participants,” and it was therefore not necessary to gain consent from each individual observed. Notes taken were of general observations of the processes undertaken and did not refer to the individuals either by name or in such detail that identification would be possible.

#### Confidentiality

All participants were allocated a unique study identification number that was used to identify them on all study records (e.g., interview recordings, transcripts, and survey responses). The link between this study number and participant names and contact details was stored in a password-protected file in a secure folder with access limited to the immediate research team. This file was only used for the purpose of sending survey invitations and reminders and to invite participants to interviews.

Participant names do not appear in any publications, and participating clinical areas are referred to by a letter code rather than by name to help maintain the anonymity of participants. Where quotes from interviews are used, these have been anonymised and a pseudonym was chosen for each interviewee – to maximise confidentiality pseudonyms do not necessarily reflect the gender, age, or ethnicity of the participant.

#### Safety monitoring

Adverse events (AEs) related to use of the spray were expected to be equivalent to those experienced with other similar cleaning products, including mild skin, eye, and respiratory irritation. All staff using the spray were directed to report AEs to the site research team, as well as completing any standard local occupational health processes. Information on AEs was collected whether volunteered by the participants during data collection or discovered by or reported to the site research team. AEs unrelated to the study were not reportable. Any related AE that met the standard criteria for seriousness was automatically deemed to be unexpected and had to be reported within 24 h of the site research team becoming aware of the event.

## Results

### Participants

In total, 182 participants were recruited to participate in the implementation study; of these, one withdrew before the end of the implementation. In total, 102 of these participants completed the first survey, 66 completed the second survey, and 23 participated in qualitative interviews. All interviews were conducted between 5 February 2021 and 26 March 2021. Individual interviewers conducted between two and six interviews each.

### Findings

The findings of the main evaluation have been submitted for publication in a relevant clinical journal. In summary, the results suggest that the spray-based disinfectant has a number of perceived advantages over existing disinfection processes, and most participants found it a positive addition to their cleaning practices. There were other factors, however, that could limit its potential utility. As well as the main evaluation findings relating to adoption of the spray, the qualitative analysis also explored more general themes relating to HCWs' experience of clinical work and infection control. This analysis explored how clinical perspectives and practises adapted as a result of the pandemic, and how this context potentially affected HCWs' ability and willingness to adopt new processes such as a novel disinfectant spray.

During the interviews, participants reflected on what they would like to see whether this or other similar sprays were to be implemented widely in the NHS. Commonly mentioned issues reflected their priorities in terms of IPC processes and their clinical working environment. First, a desire for “*evidence*” appeared to be an important factor for many: convincing evidence of the effectiveness of the spray in eliminating viruses, clear evidence of its safety, and evidence of cost-effectiveness. Adoption and endorsement of the spray as an approved product in the NHS was seen as potentially providing reassurance that these evidence requirements had been met. Second, the spray needed to “*fit*” in easily with their existing working practices. For some, this meant it needed to take less time, for instance by replacing existing cleaning rather than being an additional step, or drying more quickly, so that they could fit it into a busy cleaning schedule. Others thought the risk of adverse respiratory reactions and restricted use on medical devices were potential barriers to adoption. A third group of factors was related to “*logistics*;” for instance, some interviewees mentioned the need for a clear protocol for how to access supplies and restock, and others wanted clear guidance about where bottles will be stored in clinical environments for the ease of access and to maximise usage. Likewise, the ability to recycle bottles was appealing to many, but plans needed to be put in place for how this would happen.

### Dissemination

Emerging findings were shared during the study both within the project team and with the industry partner to facilitate implementation. The primary output of the project was a final report produced for the industry partner, which was submitted at the end of the study and informed further development of the spray and distribution strategy to facilitate adoption in the NHS.

In addition to publishing the main evaluation findings in a clinical journal, we plan to submit a further paper on HCWs' experience of IPC during the pandemic, which will be submitted to a social science journal.

## Discussion

### Doing rapid research: Practical challenges and opportunities

#### Ethical approval and set-up

**Figure 3:**
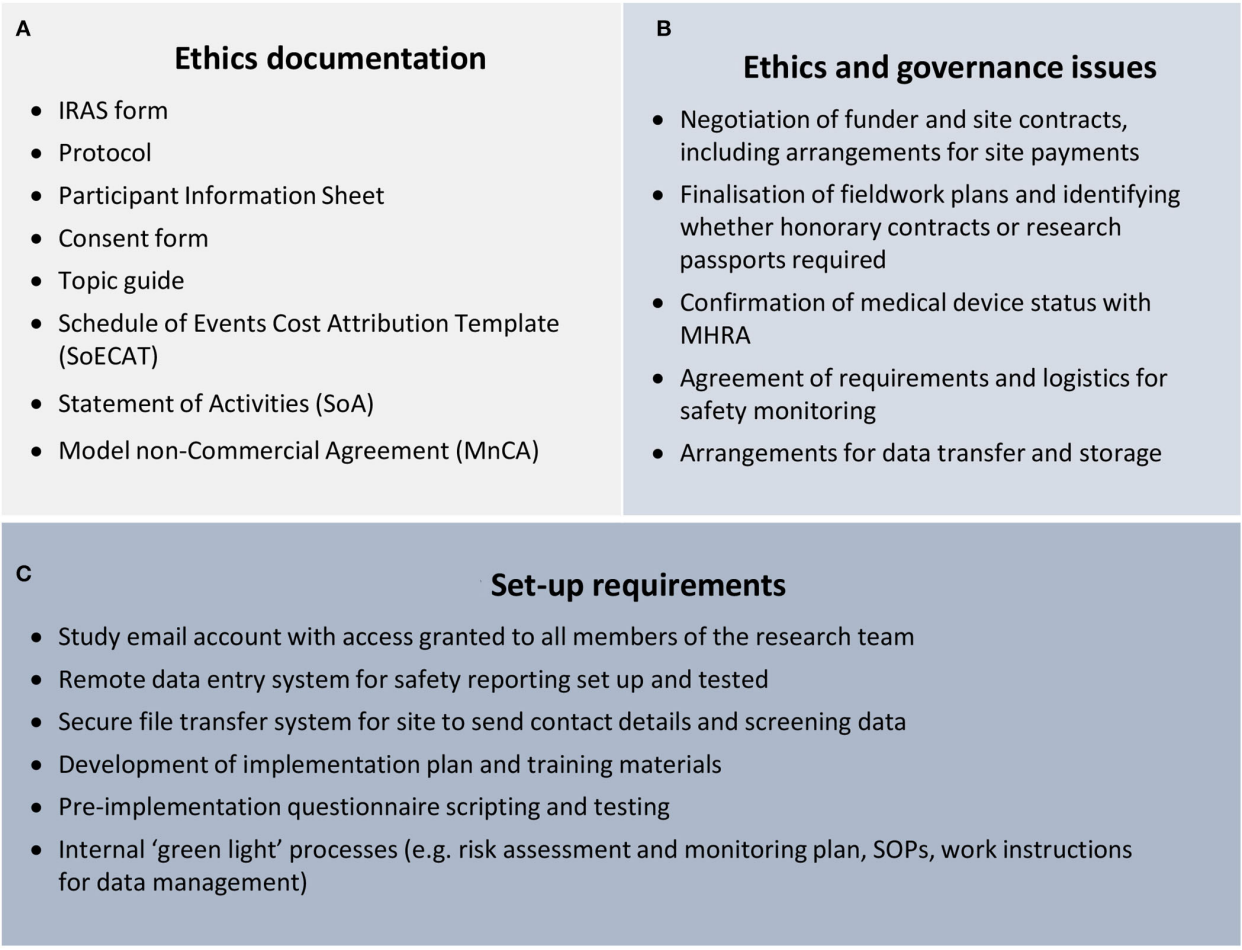
Set-up activities.

Vindrola-Padros et al. (2020b) note that there can be a preconception that rapid research will not have gone through the same rigorous ethics process as other studies, making it seem like a “*quick and dirty*” alternative to “*proper*” research. We did not experience this preconception *per se*, but there was an expectation that expedited ethics processes in place for COVID-19 studies would mean that set-up timescales would be very quick. The study did benefit from fast-track NHS REC and HRA review, as well as expedited timelines for both sponsor review and site approval. However, although the timelines for fast-track review were much quicker than for a standard application, the documentation required for the applications was not reduced (refer to Figure 3A).

Although producing a well-considered protocol and ethics application enabled the team to work through important questions of study conduct, the time required to do this was hard to minimise. There were also a significant number of issues that needed to be discussed and resolved before the ethics application could be submitted and approvals granted (refer to Figure 3B). These issues had to be negotiated with various departments, including the clinical trials unit (CTU) quality assurance team, the contracts team, the sponsor's office, and IT support. Although colleagues in these departments were extremely supportive and responded to queries as soon as possible, the backwards and forwards nature of the discussions meant that resolving each issue inevitably took a number of days, and the cumulative impact on set-up timelines was considerable.

The pressure on set-up timelines was exacerbated by the fact that as well as securing ethical and governance approvals, there were also a number of systems and processes that needed to be in place before implementation could begin (refer to Figure 3C). It was a similar situation with the shift to digital working during the pandemic, which made many study activities easier (e.g., virtual meetings, arranging, and recording interviews), but also created a number of challenges, such as researchers not having access to phone lines when working from home. It was also the first time these platforms had been used for research in our team, which meant we were learning about functionality (such as automatic transcription and anonymising recordings) throughout the early stages of the project. This was also true for our participants who had to adjust to the different dynamics of organising and attending virtual meetings and interviews. The interviewing team being based in different organisations also made setting up efficient processes more challenging; for instance, the Microsoft Teams interface worked differently when logged in from different institutions and arranging for non-university staff to have virtual access to shared folders was not straightforward.

#### Implementation and fieldwork in a pandemic

The relative complexity of the CLEAN study made planning and setting up challenging. In comparison with some other research conducted with HCWs during the pandemic (e.g., Vindrola-Padros et al., 2020a; Rücker et al., 2021; Smith et al., 2022), our study was distinctive both because it was interventional rather than purely observational (i.e., the study team was involved in delivering an intervention as well as collecting research data) and because of the variety of primary data collection methods involved (observations, online surveys, qualitative interviews, and safety data reporting). As well as complicating the set-up process, this complexity introduced the challenges during fieldwork, largely because many elements of the study were interdependent and had to happen sequentially rather than concurrently. For instance, the observations fed into implementation plan and so had to be completed before the implementation could start, the first survey had to be sent out immediately after consent but ideally before a participant started using the spray, and the qualitative interviews and second survey could only take place after participants had been using the spray for some time.

Implementation took place during the peak of the winter 2020–2021 COVID-19 wave, spanning the period when bed occupancy and pressure on staff was at a peak in the hospital. There were associated challenges in some of the clinical teams that had initially been identified for the study, delaying and in some cases preventing implementation taking place in these areas. This did, however, mean that the study was seen as extremely relevant by HCWs. The COVID-19 situation was also changing throughout the study, which had a significant impact on both implementation and fieldwork; for instance, the emergence of a new variant created questions about efficacy that had to be resolved before the spray could be used in the hospital. The study also spanned the period when HCWs received their first COVID-19 immunisations, reducing their immediate risk of severe illness although most continued to be very aware of the risk of transmission to patients and to their family and friends.

In this context, it was important that we were able to be agile and adapt our approach to respond to the challenges in the implementation setting. We took a flexible approach to engaging with staff in the clinical areas to accommodate the pressure they were under; for instance, in some cases, a one-to-one meeting was held, and in others, research team members attended clinical team meetings to explain the project. Instead of implementing the spray across all areas at once as originally planned, we staggered the implementation, so we could start recruitment in those areas that were ready as soon as possible. It was also important that we responded to the changes during the pandemic context that could have had an impact on perceptions and use of the spray. For instance, questions were added to the survey about vaccine status, as fieldwork took place during the period that HCWs received their first vaccinations, and this could have affected participants' perception of their personal level of risk.

The staggered implementation also allowed us to learn from early experiences, so we were able to adapt the implementation strategy and ensure as many staff as possible used the spray. For instance, we discovered that supply logistics meant participants in some areas were not getting access to the spray for some times after signing up to take part, so we started giving a bottle of spray to each participant when they consented, so they could start using it straight away. We also extended the implementation period beyond what had initially been planned to allow the areas that implemented later in the study enough time to provide useful feedback. This also allowed us to observe changing use of the spray over time – including it becoming routine in some teams – and to see how use changed with the shifting pandemic context. We also expanded the number of participating clinical areas when we became aware of additional environments which could potentially benefit from spray, were interested in participation, and would add to the diversity of environments in which to explore usability. These areas provided valuable additional feedback and mitigated the impact of those areas not able to implement the spray.

#### Sampling

The short time available for fieldwork and the challenges with implementation limited our ability to sample exactly as planned. We originally intended to use responses from the second survey to inform the sample for the qualitative interviews, but the delayed and then staggered implementation made this impossible, as we had to complete most of the interviews before the second survey was launched. We had also intended to use purposive sampling to select all the participants for the qualitative interviews; however, a high proportion of those we initially sampled were unable to take part before the end of fieldwork. We therefore adapted our sampling strategy to be less purposive and more opportunistic in terms of availability of participants and timing of interviews. We continued to focus on ensuring we included people from different clinical environments, but accepted that in terms of our other sampling criteria, our sample was more self-selecting than we had originally intended.

Discussions with the site recruitment team during our weekly project meetings suggested that the shifting pandemic context may have been an important factor in response rates to the surveys. In the early stages of recruitment, many staff were enthusiastic about signing up for the study, but may have been too busy to complete the first survey because of the heightened pressures caused by the new wave. However, by the end of the fieldwork period, case numbers had decreased substantially and the decreasing pressure in the hospital meant some staff – as noted by one team member – “*feel like COVID is over*.” Our multi-disciplinary team allowed us to be aware of this dynamic and better understand the perspectives of our participants. However, this resulted in a smaller survey sample than we might have expected without the additional burden the pandemic placed on our participants.

The tight study timescales precluded a significant extension to the fieldwork period, which limited our options for addressing any sampling issues caused by the lower-than-expected response rates. Furthermore, some actions we considered (such as introducing an incentive for the second survey or changing the consent form to make the optional consent to the interviews clearer) would have required an amendment to the ethical approval. Although the amendment review process would have been relatively quick, the project was progressing so fast, and it was not practical to make these changes in time for them to have an impact.

We were, however, able to take a number of other steps to address the recruitment challenges within the limited time we had available and mitigate the impact on sampling. We identified the purposive criteria that were likely to have the most impact on the experience of using the spray (clinical environment and seniority) and conducted targeted recruitment to ensure a good spread of interviews in these areas. We recruited additional environments and staff to the study to ensure that our sample included a wide range of hospital environments and health professionals, and in this respect, we met or exceeded our sampling objectives. In addition, implementation and fieldwork were extended by a month to give us longer to collect survey responses and conduct interviews, and clinical contacts at site encouraged staff to complete the surveys and flexible interview times were offered (including outside of working hours) to maximise participation. The flexibility we were able to offer for interviews was an advantage of our rapid approach, which meant we had a group of interviewers available rather than only one or two, as is common in many standard qualitative projects.

Our ability to respond quickly to the challenges and adapt our approach where necessary allowed us to largely overcome the practical challenges experienced during recruitment, although there were still some limitations in the sampling. We would have preferred to hear from more people in the second survey, as we do not know how the non-respondents would compare with respondents in terms of their experience of using the spray. The interview sample was also predominantly made up of experienced NHS professionals, who could have been more (or less) open to different cleaning methods and may have experienced the pandemic differently from less experienced staff. In addition, the majority of the interview sample described themselves as White. Given the variation by ethnicity in impact of COVID-19, this could potentially be a limitation of the analysis. Such issues with the seniority and ethnic diversity of qualitative sampling have also been experienced in other rapid research studies conducted with HCWs during the pandemic (Hoernke et al., 2021; Singleton et al., 2021).

Despite these minor limitations, we successfully recruited the participants from a range of clinical teams, professions, and ages in a very short space of time, achieving analytic saturation. There was also a significant benefit to the mixed-methods approach – for instance, we were able to include additional groups (e.g., doctors) in the qualitative interviews who were under-represented in the survey samples, and the consistency of key messages across interviews, surveys and observations, and across different hospital environments, gave us greater confidence in our findings. Considerations of data adequacy also took into account the richness and quantity of data, with analytic saturation being achieved with a much smaller number of interviews than expected. Initially, it was anticipated that interviews would be short given most took place with busy clinical staff, often during the working day. However, interviews were longer (several lasting over an hour) and richer than anticipated, with interviewers feeling participants appeared very keen both to talk about their experiences and to contribute to a study that could potentially help the hospital deal with the challenge of the pandemic. This was also reflected in feedback from the lead research nurse, who suggested many staff valued having an opportunity to talk about the challenges they had experienced during the pandemic.

#### Saturation in rapid qualitative analysis

Our reflective discussions regarding sampling raised some issues around when to cease data collection in a rapid research. The concept of saturation in qualitative research more widely has been increasingly examined, its use critiqued, and alternative concepts, including information power, proposed (Malterud et al., 2016; Saunders et al., 2018; Sim et al., 2018; Braun and Clarke, 2021). Saunders et al. (2018) identify four different variants of saturation; data, thematic (in two forms), and theoretical. None of these resonate fully with our rapid qualitative analysis. We initially applied the term “data saturation;” however, other authors have used this to describe a process where data collection is separated from and precedes detailed analysis. By contrast, our analysis involved an iterative process where data collection and analysis were carried out concurrently during an intense and immersive period of engagement with our topic and setting. Both thematic and theoretical saturation seem to emphasise the communication of the analysis – the themes or theory that is developed. With our rapid analysis, although we sometimes used the term “theme” to refer to categories which summarised something important in our analysis, the aim was less about presenting a well-developed *concept* – a theme or theory – and more about the overall storey that we needed to tell about the potential value of this new intervention in the context of the pandemic. Themes and theories can be the powerful tools for communicating an analysis, but they take time to mould. Moving beyond descriptive categories to themes that have resonance beyond the immediate study may be challenging for researchers who do not have experience of qualitative research and social science.

Our decision about when to cease data collection was driven by an assessment as to whether we had “adequate data to tell a rich, complex and multi-faceted storey about patternings related to the phenomena of interest” (Braun and Clarke, 2021, p. 211 referring to Sim et al., 2018). Notwithstanding Braun and Clarke's criticism of the concept, “saturation” seemed an appropriate shorthand for this. Aspects relating to information power (Malterud et al., 2016) did enter our decision-making – for example, we discuss the unexpected richness of our data. However, information power suggests to us a quality that resides more in the data – whereas the concept of saturation conveys something important about the extent to which the team have been able to use the data to develop their analysis. We also feel that information power is harder to directly apply in terms of making a decision regarding whether to cease data collection – yes the data are rich – but is it “enough?” In the end, we have used “analytic saturation,” which puts the emphasis on the analysis rather than the data, but reflects that our output was primarily a storey, albeit with sections and headings, rather than themes. This is an important topic within rapid qualitative research, which warrants further consideration.

### Analysing rapid research: Getting the most from the data

#### Rapid analysis procedure sheets

From the outset of the project, we were aware that the condensed timeline, combined with delays in study set-up, would apply pressure to the analysis. To mitigate this time pressure, we began analysing data as soon as it was collected through the use of RAP sheets and subsequent reflective discussions at weekly analysis meetings. We began by aggregating thematic data from each interview into one RAP sheet (as described by Vindrola-Padros et al., 2020b); however, our early analysis discussions suggested that it would be beneficial to keep a clear link to the individuals. We therefore modified the approach, completing individual RAP sheets for each interview and maintaining a master RAP sheet to draw together emerging themes. This was similar to the process used by Gale et al. (2019), although our RAP sheets were completed from memory straight after the interview rather than as a summary of the full transcript, and involved a combination of factual interview content and interpretative reflections from the interviewer.

Our approach allowed for the easier identification of differences between interviewees and clinical environments, made it easier to record specific examples of issues to refer to during analysis, and gave us confidence in reaching “meaning” as well as “code” saturation (Hennink et al., 2017). It may be that this level of detail is particularly important for the studies such as ours with relatively applied objectives, which many rapid research studies tend to be. We also found our approach simpler, making it quicker and easier to complete the RAP sheet after each interview. The synthesis of data and defining of themes/sub-themes became a team activity, rather than each individual researcher synthesising their data as they progressed through the interviews. Simplifying the process was particularly beneficial, given the range of experience within the interviewing team. Aggregating data in real time can be quite demanding even for experienced qualitative researchers; it is even more challenging for researchers with limited previous experience of thematic analysis.

Retaining the link to individual cases was particularly important for our analysis because full interview transcripts could not be produced in time for the final report, and the use of individual RAP sheets facilitated conducting the analysis without the full transcripts being available. Each interviewer used either the audio recordings and/or the automatic transcription produced by Microsoft Teams to retrieve specific quotes from their recordings to illustrate key themes for the final report. As similar focussed approaches have been found to yield results comparable to analysis of full transcripts (Vindrola-Padros and Johnson, 2020, p. 1600), this approach appeared to be suitable for a rapid analysis such as ours, with plans for subsequent secondary analyses once full transcripts become available.

We will also further explore the potential of automatic transcription to speed up the production of transcripts for future rapid studies, although a significant amount of time would still be required for checking, editing, and anonymising the transcripts. It is also important to take into account the time required to analyse full transcripts in comparison with the more focussed approach we adopted, and the potential differences between automatic and manual transcriptions would need to be considered (Vindrola-Padros and Johnson, 2020).

#### Oral analysis process

Our approach to RAP sheets facilitated an early synthesis of key data without requiring significant analytical input. This was complemented by weekly analysis discussions where we had the opportunity to identify and develop themes and to reflect more analytically on the data. This oral analysis process facilitated constant comparison across cases. Individual researchers had a good knowledge of the interviews they had conducted, supported by the RAP sheet which focused attention on the key information from that case. During the weekly meetings, individual researchers made observations based on their cases, and tentative interpretations could be developed immediately with reference to other cases. For example, when one interviewer observed that the small bottles were preferred by their interviewees, the other interviewers could support or refute that observation based on their cases. The team could then develop working hypotheses (e.g., “small bottles are preferred by teams working across different environments” and “larger bottles are preferred in office-based environments”), which could immediately be tested, explored, and developed in subsequent interviews.

As well as facilitating constant comparison, the weekly analysis discussions provided a number of other important benefits for our rapid approach. Weekly meetings allowed all the members of the large interviewing team to participate in the analysis and contribute to the developing thematic structure, and by discussing their observations and the similarities and differences across interviews, all researchers developed a good understanding of the entirety of the dataset. The academic researchers also had the opportunity to clarify initial interpretations with the junior doctors who worked in the implementation environment and were familiar with the use of the spray, providing many of the same benefits as formal member checking (which would not have feasible in the timescales). This oral analysis process may also have been more accessible for the less experienced researchers in the team than a traditional transcript and text-based qualitative analysis process, as it enabled them to work alongside more experienced researchers and benefit from their experience while still being able to contribute to the analysis. As such, our rapid analysis approach using simplified RAP sheets and oral analysis sessions could be particularly beneficial for studies using peer researchers, whom it can sometimes be difficult to involve meaningfully in the analysis process (Powell et al., 2021).

The use of theory was another important factor in our rapid analysis strategy. The study design, interview topic guide, and survey content were all informed by the NASSS and NPT frameworks, which were also reflected in the categories used to summarise the key findings on RAP sheets. The qualitative analysis, however, was largely inductive, using themes that emerged from the data rather than applying an a priori theoretical framework. We considered inductive analysis to be most appropriate for qualitative research that aimed to understand the experiences and perspectives of interviewees, especially in a new social environment (the pandemic) and with an untested intervention (implementing the new spray in an NHS context).

The inductive analysis of the qualitative data combined well with the deductive approach of the surveys to allow us to assess the intervention in the light of both theoretically driven concepts and participant driven experiences. This approach allowed us to quickly understand the implementation context and identify the potential benefits and drawbacks of the spray, which addressed our specific research questions. Another option would have been to apply a more deductive theoretical framework in the initial analysis (such as the process used by Nevedal et al., 2021). This could potentially have made it easier to quickly locate our findings within wider academic discussions; however, this kind of purely deductive analysis can create pressure to “force” qualitative data into an inflexible framework (May et al., 2018).

#### Mixed-methods and secondary analysis

Data from observations, surveys, and interviews were being integrated throughout the study; for instance, observational data informed the implementation plan, and early analysis of interview data informed the design of the post-implementation survey. However, the compressed reporting timeline made it challenging to conduct a thorough, comprehensive mixed-methods analysis. Although our oral analysis discussions did explore the links between emerging quantitative and qualitative findings, these results were largely analysed independently because these report sections needed to be written concurrently to deliver actionable findings quickly. This gave us limited scope to fully “follow the thread” (Alexander et al., 2008) between the various data sources, as we normally would in a mixed-methods analysis.

It was also challenging to incorporate non-interview data in the final analysis (e.g., notes from the site recruitment team, feedback from observations, and meeting discussions about implementation). This was valuable information that fed into many aspects of the implementation and data collection, as well as being used informally during our analysis discussions, but it was hard to document and incorporate into the analysis in a more formal and systematic way due to the time pressure. Overall, each method of data collection contributed useful insight which helped to address the research questions, but the limited opportunity for integrating data from different sources meant that although the analysis was robust and informative, it was not as rich or nuanced as it could have been had there been more time available.

Overall, the limited time available for analysis meant we had to be very targeted in our approach, which largely focussed on addressing the immediate objectives of the study. This would not necessarily be suitable for all research questions, but it was appropriate for our project given the specific nature of the research questions. It allowed us to deliver actionable findings in a very short space of time, but did not allow us to fully explore the richness of the data or maximise the value of the mixed-methods approach. One way to address this would be to use rapid analysis to deliver the main research findings as quickly as possible and then conduct secondary analyses at a later date when there is less time pressure, full transcripts are available, and data from all sources can be examined together. Secondary analysis would also provide an opportunity for applied rapid findings to be revisited through different theoretical lenses – for instance, recent papers have re-examined data from rapid research in the context of boundary work (Vindrola-Padros et al., 2021b), gender (Regenold and Vindrola-Padros, 2021), and the sociology of emotion (Dowrick et al., 2021).

### Rapid research with a multi-disciplinary team

#### Benefits of a multi-disciplinary team

The range of expertise within the project team was one of the most important factors enabling us to undertake the CLEAN study in a short space of time. The study was coordinated by Leeds Institute for Clinical Trials Research (LICTR) and the NIHR Surgical MedTech Co-operative. It was delivered in collaboration with Leeds Teaching Hospitals Trust (LTHT), and the spray manufacturer was an industry partner on the grant. The project was delivered by a multi-disciplinary team based across UoL and LTHT, jointly-led by AQ (Associate Clinical Professor of Surgery at UoL/LTHT) and NR (University Academic Fellow in Healthcare Technology Evaluation at UoL). The research team consisted of NR along with two other experienced qualitative/mixed-methods researchers (SP and RH) and three junior doctors (RS, MK, and JH). Recruitment and implementation at site was overseen by AQ and led by a Senior Research Nurse at LTHT. IPC expertise was provided by NY (Consultant in Medical Microbiology and Lead Infection Control Doctor, LTHT) and PL (Consultant in Infectious Diseases with special interest in new and emerging infections and Speciality Lead for Infectious Diseases and High Consequence Infectious Diseases, LTHT). Senior clinical and academic oversight was provided by a Professor of Surgery at UoL/LTHT and a Professor of Clinical Trials Research in LICTR. Study management, quality assurance, data management, and IT support were provided by LICTR.

The involvement of key stakeholder groups, in particular strategic decision-makers for infection prevention and control in the hospital, was an important factor in the feasibility and overall success of the implementation. The site research team responsible for recruitment, training, and implementation were also heavily involved in the design and management of the study, which facilitated a feasible, flexible, and responsive approach and was crucial for obtaining approvals quickly. Regular meetings between the project team and the industry partner were helpful in providing early access to samples of the product and to findings from a simulation study, which facilitated the development of the training and implementation plan.

The junior doctors who conducted observations were all carrying out clinical duties in one of the clinical areas participating in the study. Importantly, this provided an essential link to the implementation environment for the social scientists within the team, who were unable to attend the project site because of COVID-19 restrictions. It was invaluable to have team members with direct experience and knowledge of the clinical environments and challenges being faced in the hospital helping to direct data collection and facilitate access. The fact they were already based on site meant no research passports or honorary contracts were required, making the approvals process simpler.

The junior doctors were also involved in conducting qualitative interviews, which doubled the size of the interviewing team and made it possible to undertake a much larger number of interviews in the short space of time available and flexibly accommodate the schedules of busy participants. We also found the combination of academic and clinical researchers in the team introduced different perspectives and interviewing techniques that complemented each other and strengthened the research. The experienced qualitative researchers tended to be more exploratory during interviews, and more likely to notice and explore wider themes, whereas the doctors were quickly able to elicit clear, relevant information from interviewees and recognise productive avenues to probe further because of their familiarity with the field. It was also very useful to have these different, complementary perspectives contributing to the analysis discussions and member checking, with the qualitative researchers being able to synthesise ideas and generate themes quickly and the doctors being well placed to make sense of the findings in context and to offer alternative explanations for observed patterns in the data.

There were also benefits for the doctors for their own professional development. They gained training and experience in qualitative research methods and found this helped them adapt their approach to interviews, which was usually fairly rigid as a result of medical history taking that is normally quickfire and specific rather than semi-structured and exploratory. There is rarely the opportunity to participate in this type of research during medical training and it will be a beneficial skill in their future practice, especially with the modern clinical research focus on patient-reported outcomes. Gaining experience of a multi-disciplinary team at work and being involved in study group meetings was also valuable experience as this is not something the trainees are necessarily exposed to in their NHS roles. Although the time demands of the rapid study were challenging, the rapid timescales also facilitated their involvement, as it meant they were able to experience the whole research process, from design and planning to fieldwork, analysis, and reporting. Because of short clinical placements, this might not have been practical in a longer study.

#### Practical challenges

The doctors' involvement was crucial to the delivery of the project and the robustness of the analysis and findings. There were also challenges involved, however, perhaps the most important being the difficulty for the doctors of balancing their clinical roles with attending training sessions, conducting interviews, and contributing to the analysis. Clinical academics usually have a form of day release or small blocks for research between out-of-hours clinical commitments, so this was sometimes challenging to align with university working hours. There were also logistical challenges associated with delivering training and coordinating the work of such a large group of interviewers; although the increased size of the team did make it possible to do the research in a shorter timeframe, it did not necessarily result in a net saving of working hours for the academic researchers. The size of the interviewing team (in combination with the limited time and lack of full transcripts) also meant that we did not become as familiar with the raw data from all the interviews as usual, although the rapid oral analysis process mitigated this to a large extent.

We overcame these challenges in a number of ways, including holding regular virtual meetings to discuss progress and share different analytical perspectives, developing SOPs for interview conduct to ensure everyone was working to the same processes, and using shared documents such as interview logs and RAP sheets to facilitate collaboration, project management, and oversight. Flexibility from all team members helped us to coordinate regularly, even if not everyone could attend every meeting, and with multiple clinicians on the team, we were normally able to cover for each other when needed. We also aimed to provide training for the doctors that was targeted and efficient, given the other demands on their time, but that was also comprehensive and practical enough that they felt confident to undertake their interviews.

#### Team-based reflexivity

**Figure 4:**
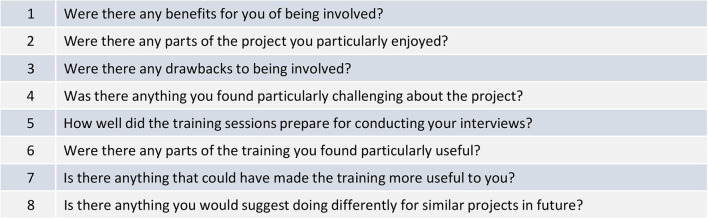
Examples of orienting questions.

Rankl et al. (2021) propose a model for incorporating team-based reflexivity in rapid research, which includes dedicated time during regular meetings for the team to reflect on progress and informal individual reflexive discussions (underpinned by orienting questions). We incorporated these elements in our study in three main ways: (1) weekly project meetings which were conducted throughout the study and attended by the whole project team); (2) weekly analysis meetings attended by the interviewing team which were conducted during recruitment, fieldwork, and analysis; and (3) individual reflexive discussions between members of the interviewing team, conducted verbally and/or by email between the interviewing team during the writing of this paper (Figure 4 shows the examples of orienting questions used during these discussions). These activities were supplemented by the qualitative training sessions provided for the junior doctors conducting interviews and also shared channels on Microsoft Teams where all team members could post updates, queries, and links to useful information as well as discuss any emerging issues in between meetings.

The weekly project team meetings and shared Teams channel were invaluable in allowing the site team to discuss their experiences of implementation and recruitment, helping us to identify the problems early and implement solutions quickly. These structured mechanisms for the team to reflect on progress were a crucial factor in enabling us to be responsive and “think on our feet,” which was much more important in this rapid study than we have found during other more standard qualitative fieldwork. Regular team-based reflection also provided the University-based researchers with valuable insight into the implementation context in the hospital, for instance the potential reasons for low response rates and the possible benefits to staff of participating in qualitative interviews which were discussed earlier in this paper. This was particularly important because the University-based researchers were not able to access the site due to the pandemic. It helped us plan the implementation and fieldwork effectively, understand and address recruitment challenges, prepare appropriately for interviews, and contextualise the emerging analysis. In addition, notes from the weekly meetings and virtual discussions on teams provided a record of all the issues experienced and actions taken during implementation and fieldwork, as well as team members' feedback, reflections, and suggestions, all of which formed an important part of the analysis.

The weekly analysis meetings and initial qualitative training sessions provided further opportunities for reflecting on progress, allowing team members involved in the interviewing to discuss their experiences and for the less experienced junior doctors to prepare for their interviews and then reflect on their progress as they began to accumulate more experience. These meetings were also instrumental in elucidating and taking advantage of the diverse experiences and perspectives within the team.

Self-location was an important aspect of these discussions, especially given the diverse nature of the interviewing team and their varying relationships to the fieldwork setting. The junior doctors in particular had multiple roles within the study; as implementers, evaluators, and a link between the academic researchers and the site, but also to some extent as members of the study population. Their insider perspective (Merton, 1972) was extremely valuable both practically, as discussed above, and methodologically, as their familiarity with the setting helped the academic researchers to interpret, contextualise, and validate the emerging results. However, they will inevitably have had preconceptions of both the setting and intervention shaped by their own experiences, which will have influenced attitudes towards the research to some extent. To address the potential risk that the clinical interviewers would not explore interviewee responses in detail because of a shared understanding of the situation and context, the interview training focused particularly on probing and unpicking what might initially be “taken for granted.”

It is also relevant that although the junior doctors can to some extent be considered “peer researchers,” the majority of the interviewees were experienced nurses, making the insider status of the doctors as researchers more a point on a continuum rather than a stable, fixed identity (Hellawell, 2006). The relative professional identities attached to these differing clinical roles and levels of seniority are therefore likely to have been a factor in the interpersonal dynamic during their interviews. In some cases, this may have been beneficial, for instance in creating rapport and having a common understanding of the issues being discussed, but it may also have been detrimental at times.

In contrast, interviews conducted by the academic researchers will have been shaped by their “outsider” status – for instance, some interviewees may have been happier to share certain things with someone seen as being neutral, but others might have found it harder to explain their experiences to someone with no experience of the environment they worked in (Bridges, 2001). It could also be difficult hearing frontline staff describe the day-to-day challenges they faced during the pandemic, and for the academic researchers, this was a stark reminder of how harrowing the situation had been in the hospital at times. As outsiders, this made us particularly aware of the need to approach interviews with empathy and sensitivity, and this may to some extent have affected our approach – for instance, occasionally feeling limited in our ability to probe on certain issues to avoid appearing judgemental of a situation we had not had to face. It also made us even more conscious than usual of our responsibilities to the research participants, both in terms of avoiding placing any unnecessary burden on them and also doing our best to ensure that the research they gave up their time for was valid, robust, and useful.

Other authors have highlighted the risks of a lack of cohesion or conflict in insider/outsider teams (Louis and Bartunek, 1992; Durand Thomas et al., 2000). This was not something that we experienced – rather we were surprised at how quickly and effectively a cohesive team was formed, particularly given that we never met in person during study design or conduct. The pandemic context and COVID-19 focus of the research may have played a part in this. However, it may also be the case that the short but intense nature of rapid research projects facilitates team cohesion compared with projects where relationships have to be sustained over a longer period.

Overall, the benefits of our multi-discipline team far outweighed the challenges it created, although it is still important to be aware of these challenges and to plan accordingly when conducting multi-disciplinary rapid research. In particular, it is important to be mindful of the additional pressures that being involved in rapid research, either as a participant or as a researcher, could place on HCWs, particularly during periods (such as future waves of a pandemic) when they are already facing considerable additional pressures. Regularly, structured team-based reflection and reflexivity helped us to ensure our study was sensitive to these pressures. Dedicated time for the team to reflect on progress meant that our fieldwork plans were feasible, potential problems were recognised and addressed early, and researchers felt supported. It also allowed us time for considering our own self-location and how each researcher's personal experiences and perspectives have shaped the research.

## Conclusion

The very conditions that make rapid research necessary can also make it extremely challenging to undertake. Conducting fieldwork and analysis quickly inevitably requires methodological trade-offs, and there is a risk that in the process, the benefits of rich mixed-methods data, theoretical insight, and new digital technologies may not be fully realised. To combat this, it is useful to understand the aspects of a rapid study that could make it particularly challenging, so that projects can be planned to accommodate and mitigate these challenges as far as possible. In our experience, the combination of implementing an intervention, multiple data collection methods, and the pandemic setting posed various challenges during set-up, fieldwork, and analysis. These challenges can be overcome or mitigated through a combination of methodological flexibility, adapted rapid analysis techniques, digital solutions, and secondary analyses to complement and extend the initial rapid analysis.

Perhaps most importantly, knowledge of and access to the implementation and fieldwork setting was key to the success of our rapid study. There were significant benefits to having key stakeholders closely involved throughout the project and an interviewing team which blended academic research experience and familiarity with the clinical context. Although the involvement of stakeholders who are not experienced qualitative researchers in the process of qualitative data collection and analysis can be challenging, there are also significant benefits. In rapid analysis, the benefits are greatest in terms of additional input to data collection; trouble-shooting research obstacles; rapid access to key contextual information and sense checking the developing analysis. Rapid analysis methods may be more accessible to inexperienced qualitative researchers (when working in a team alongside experienced qualitative researchers) than traditional thematic methods, facilitating and enabling meaningful involvement in the analysis process.

The rapid methods employed on the CLEAN study are likely to be most useful for studies such as ours, which address relatively focussed and applied research questions and where answers are needed quickly in a rapidly changing environment. More detailed, exploratory or theoretical research aims might be more challenging to address with this type of approach, although these could potentially be explored using subsequent secondary analyses. In our experience, rapid methods are likely to be easiest to implement in settings which at least some members of the research team have direct experience of (and access to), and rapid methods may in fact be particularly useful for facilitating the involvement of such insiders in the research process. Successful delivery of relatively complex, mixed-methods rapid studies such as CLEAN is likely to require the close involvement of key stakeholders throughout the research and a large and flexible team of interviewers, supported through standardised processes, digital technology, and regular communication.

Overall, our experience demonstrates that despite numerous practical challenges, it is possible for mixed-methods research to be both rapid and robust, generating timely, targeted results to inform urgent healthcare decisions while also providing the opportunity for more exploratory, theoretically informed analysis that can make a valuable contribution to wider academic discourse.

## Data availability statement

The datasets generated for this study are available on request to the corresponding author.

## Ethics statement

The study was reviewed and approved by the Frenchay NHS Research Ethics Committee (REC). All participants provided their written informed consent to participate in this study.

## Author contributions

NR, AQ, SP, RH, PL, and NY planned, designed, and oversaw the study. RH, SP, NR, RS, JH, and MK conducted interviews, participated in the oral analysis discussions, and contributed to the team-based reflexivity. RH drafted the first manuscript version. All authors helped to review and refine the final version.

## Funding

The study was funded by the Innovate UK (Grant reference: 77807).

## Conflict of interest

The spray manufacturer (Pritchard Technologies Ltd) was an industry partner on the grant and provided supplies of the spray for the study at no cost. The industry partner took no part in data collection or analysis. The authors declare that the research was conducted in the absence of any commercial or financial relationships that could be construed as a potential conflict of interest.

## Publisher's note

All claims expressed in this article are solely those of the authors and do not necessarily represent those of their affiliated organizations, or those of the publisher, the editors and the reviewers. Any product that may be evaluated in this article, or claim that may be made by its manufacturer, is not guaranteed or endorsed by the publisher.
