# Supplementary material for: Rapid qualitative analysis in a mixed-methods evaluation of an infection prevention intervention in a UK hospital setting during the COVID-19 pandemic: A discussion of the CLEAN study methodology
Source: Front Sociol. 2022 Oct 25;7:958250. doi: 10.3389/fsoc.2022.958250 (PMC9640776; doi:10.3389/fsoc.2022.958250)
Supplement: Supplementary file 1 [file Data_Sheet_1.PDF]

frontiers

Supplementary Material

Appendix A: Observation Pro-forma

The purpose of this observation period is to observe current cleaning practice by all staff in the chosen clinical area and allowing the research team to see where, when and how VIRUSEND can be incorporated into this practice.

The proforma will act as a rough guide/ reminder for the research team and ensure observations are noted in a structured manner.

Observer: ……………………………………….

Clinical Area: ………………………………….

Date: ……………………… Time: …………………… Duration: …………………

Patient areas assessed (think about bed space, patient discharge, soiled areas, equipment, trolleys,):

…………………………………………………………………………………………………
…………………………………………………………………………………………………
…………………………………………………………………………………………………
…………………………………………………………………………………………………

Staff areas assessed (think about staff rooms, locker rooms, changing rooms, doctor's office, nurses station, ward clerk desk space, seminar rooms etc…):

…………………………………………………………………………………………………
…………………………………………………………………………………………………
…………………………………………………………………………………………………
…………………………………………………………………………………………………

Other assessments (touch points, Visitors to area, Hand hygiene):

…………………………………………………………………………………………………
…………………………………………………………………………………………………
…………………………………………………………………………………………………
…………………………………………………………………………………………………

Further comments:

…………………………………………………………………………………………………
…………………………………………………………………………………………………
…………………………………………………………………………………………………
…………………………………………………………………………………………………
…………………………………………………………………………………………………

Appendix B: Interview topic guide

<u>CLEAN: Interview Topic Guide for Health Professionals</u>

Note: *The interview schedule is developmental. The questions will need to be tailored to the specific answers of each interviewee. The interview schedule given here is therefore a general topic guide for the one-to-one qualitative interviews.*

Welcome and Introduction
Introduce self and project. Discuss confidentiality and recording. Ask if any questions. Reiterate that can stop any time. Check still happy to consent.

Re-cap of Research and Plan for Interview
Brief re-cap on the aims and purpose of the interview and explain what will happen.

The following questions need not be covered in this particular order but rather the interview should flow as freely and naturally as possible. The interviewer will prompt as appropriate with phrases such as 'can you tell me a little more about that', 'can you give me an example of that', 'how did/do you feel about that'.

Question: Can you tell me a bit about your clinical career?

- Role
  - Has this changed with COVID-19?
- Length of time at LTHT
- Clinical environments currently work in

Context – experiences of infection control

- What has been your experience of infection control processes under COVID-19?
  - What are the usual COVID-19 procedures in [clinical environment]
  - What are your specific responsibilities within this?
  - What has changed from what was done pre-COVID?
    EXPLORE: Hand washing/sanitising
  - What has changed during COVID-19 pandemic?
  - What has worked well/less well?
  - How confident are you that existing processes are adequate to minimise transmission of COVID-19 within [clinical environment]?
  - Are you aware of any current gaps where you worry transmission could occur?

  Explore:
    - contaminated surfaces vs airborne transmission
    - First wave vs second wave

Views on VIRUSEND - preparation

- How did you hear about the VIRUSEND study?
- How do you feel about LTHT taking part in the VIRUSEND study?
    - Research during COVID-19
- What training have you received in the use of VIRUSEND?
- Were you clear after the training about how and when to use VIRUSEND?
    - Check – clarity of safety and efficacy messaging – how confident do they feel about this?
- Was VIRUSEND what you expected?

Experiences with VIRUSEND

Are you aware of VIRUSEND being used in [clinical environment]?
Have you used VIRUSEND?

- Why/Why not
- How often
- Where and when

What do you think of VIRUSEND?
How does using VIRUSEND compare with your usual infection control measures?

- Pros and cons

Have there been any problems with using VIRUSEND?

Explore:

- Practicalities – location of VIRUSEND when needed
- Speed and ease of use
- Pros and cons of spray vs wipes etc
- Any worries/adverse events

- How does VIRUSEND fit with your normal infection control measures?
- How does VIRUSEND fit with established infection control pathways?

What do your colleagues think of VIRUSEND?

Would you continue to use VIRUSEND if it was available after the study?

- Explore – cost - existing wipes and chlorine based system is cheap – are any VIRUSEND benefits worth the expense?

Are there other clinical environments where you think VIRUSEND would be useful?
What messages would you pass on to other trusts considering buying VIRUSEND?

Anything not covered?

- Anything else you'd like to add about your experiences of the study?

Closing and Thanks

Conclude the discussion and thank the participant for their time and contribution.

Appendix C: RAP sheet template

Summary of main interview content	Emerging ideas, themes
**Clinical work during COVID**	
**Experiences of IPC during COVID**	
**Views on VIRSUEND**	
**Experience of using VIRSUEND**	
**View on using VIRSUEND in the future**	
**Other**	
